# Supplementary material for: Analysis of PPARγ Signaling Activity in Psoriasis
Source: Int J Mol Sci. 2021 Aug 10;22(16):8603. doi: 10.3390/ijms22168603 (PMC8395241; doi:10.3390/ijms22168603)
Supplement: Supplementary file 1 [file ijms-22-08603-s001.zip › Supplemental materials_Analysis of PPARg signaling activity in psoriasis/Pathway models/Models images and html files/Interleukin-17 and interleukin-22 signaling in psoriasis/9321.html]

MAPK3


# Protein MAPK3

|  |  |
| --- | --- |
| URN | urn:agi-llid:5595 |
| Total Entities | 0 |
| Connectivity | 6205 |
| Name | MAPK3 |
| Description | mitogen-activated protein kinase 3 |
| Notes | The protein encoded by this gene is a member of the MAP kinase family. MAP kinases, also known as extracellular signal-regulated kinases (ERKs), act in a signaling cascade that regulates various cellular processes such as proliferation, differentiation, and cell cycle progression in response to a variety of extracellular signals. This kinase is activated by upstream kinases, resulting in its translocation to the nucleus where it phosphorylates nuclear targets. Alternatively spliced transcript variants encoding different protein isoforms have been described. [provided by RefSeq, Jul 2008] |
| Primary Cell Localization | Cytoplasm |
| Class | Protein kinase |

---

|  |  |
| --- | --- |
| Pathway | Osteoarthritis |
|  | Skin Aging |
|  | Melatonin Effects on Circadian Cycle |
|  | High Fat Diet Activates cAMP Related Exocytosis in Synapse and Endocrine Cell |
|  | Leptin and CCK8 Activate Nodose Ganglia Neuron |
|  | Insulin Influence on Protein Synthesis |
|  | Insulin Synthesis in beta-Cell |
|  | Dopamine Receptors Signaling |
|  | Serotonin Receptors Signaling |
|  | Thyroid Hormones Common non-Genomic Effects in Hyperthyroidism |
|  | Norepinephrine Release Regulation |
|  | CRH Secretion Regulation |
|  | Insulin Signaling |
|  | Prolactin Production |
|  | Thrombopoietin Receptors Signaling in Platelet Maturation |
|  | Mineralcorticoids Secretion Regulation |
|  | T-Cell Dependent B-Cell Activation |
|  | T-Cell Independent B-Cell Activation |
|  | V(D)J Recombination Activation |
|  | Natural Killer Cell Activation through C-Type Lectin-like Receptors |
|  | Natural Killer Cell Activation through ITAM-Containing Receptors |
|  | Natural Killer Cell Activation through ITSM-Containing Receptors |
|  | T-Cell Maturation (Hypothesis) |
|  | Natural Killer Cell Activation |
|  | CD8+ T-Cell Activation |
|  | Th1-Cell Differentiation |
|  | Treg-Cell Differentiation |
|  | Th2-Cell Differentiation |
|  | T-Cell Receptor Signaling |
|  | CXC Chemokine Receptor Signaling |
|  | CC Chemokine Receptor Signaling |
|  | Plasmin Effects in Inflammation |
|  | Platelet Activation via Adhesion Molecules |
|  | Proplatelet Maturation |
|  | Platelet Activation via GPCR Signaling |
|  | Toll-like Receptor Independent Sterile Inflammation |
|  | Lipoxin A4/FPR2-Related Neutrophil Depression |
|  | Hypothalamic-Pituitary-Adrenal Axis in Acute Phase Response |
|  | MC1R in anti-Inflammatory Signaling |
|  | Eicosanoids in Myeloid Cell Activation |
|  | Leukotriene Effect on Airway Smooth Muscle Response |
|  | Neutrophil Activation via LTB4/Polyisoprenyl Phosphate Signaling |
|  | Eosinophil Survival by Cytokine Signaling |
|  | Eosinophil Activation |
|  | Mast-Cell Activation without Degranulation through CYSLTR1/CYSLTR2 Signaling |
|  | Mast-Cells De Novo Synthesized Mediators via IgE Independent Signaling |
|  | Macrophage M1 Lineage |
|  | CD16/CD14 Proinflammatory Monocyte Activation |
|  | Macrophage M2-Related Phagocytosis |
|  | anti-Inflammatory Function of Macrophage M2 Lineage |
|  | Neutrophil Recruitment and Priming |
|  | Neutrophil Activation via FCGR3B |
|  | Neutrophil Chemotaxis |
|  | Neutrophil Activation via Adherence on Endothelial Cells |
|  | Vascular Smooth Muscle Cell/Pericyte Migration and Proliferation |
|  | Vascular Endothelial Cell Activation by Blood Coagulation Factors |
|  | Vascular Endothelial Cell Activation by Growth Factors |
|  | Nociception Expression Targets Signaling |
|  | Vascular Motility |
|  | NPY1R -> CRH/POMC Production |
|  | NTSR1 Expression Targets |
|  | EDNRA/B -> Vascular Motility |
|  | ADRA1 -> Vasoconstriction |
|  | ADRA1A -> IL6 Production |
|  | ADRA2C/ADRB2 -> Vasoconstriction |
|  | ADRB1 -> Prostaglandin Generation |
|  | ADRA1 -> Prostaglandin Generation |
|  | BDKRB1/2 -> Prostaglandin Generation |
|  | CNR1/2 -> IL1B/2/4/6/10 Production |
|  | DRD2 Expression Targets |
|  | DRD1/5 Expression Targets |
|  | GALR1/2/3 -> POMC/NPY Production |
|  | HRH2/4 -> IL6/10 Production |
|  | CHRM1 -> IL2 Production |
|  | CHRM1/2/3 -> Vascular Motility |
|  | OPRK -> Pain Perception |
|  | PTGIR -> IL6 Production |
|  | PTGER1/4 -> Vascular Motility |
|  | PTGER2/3 -> Inflammation-Related Expression Targets |
|  | HTR1 -> IL6 Production |
|  | HTR7 -> IL6 Production |
|  | HTR5 -> TNF Production |
|  | IL1B Expression Targets -> Nociception |
|  | CHRNA7 -> NOS1 Production |
|  | GABA(B)R -> Postsynaptic Inhibition |
|  | NMDA Receptors -> Ca2+/CREB Activation/PGE2 Synthesis |
|  | P2RY11/13/14 -> IL8/10 Production |
|  | Luteinizing Hormone in Oocyte Meiotic Resumption |
|  | Melatonin in Cell Survival and Antioxidant Response |
|  | Circadian Clock Genes in Suprachiasmatic Nuclei Neurons |
|  | non-Genomic Rapid Actions of Vitamin D in Vitamin D Biology |
|  | Cell Cycle Overiew |
|  | Gap Junction Assembly |
|  | Scavenger Receptor OLR1 in Inflammation Related Endothelial Dysfunction |
|  | Th1-Cells Activation and Proliferation in Atherosclerosis |
|  | Proteins Involved in Dilated Cardiomyopathy |
|  | Proteins Involved in Hypertrophic Cardiomyopathy |
|  | Cardiomyocyte Hypertrophy |
|  | Hypertrophic Cardiomyopathy |
|  | Proteins Involved in Arterial Hypertension |
|  | Endothelin Signaling in Arterial Hypertension |
|  | Arterial Hypertension |
|  | Aldosterone Synthesis Acute Regulation |
|  | Endothelial Cell Dysfunction in Arterial Hypertension |
|  | Aldosterone Synthesis Chronic Regulation |
|  | Smooth Muscle Cell Dysfunction in Arterial Hypertension |
|  | TRPM4/6/7/8 Signaling in Arterial Hypertension (Hypothesis) |
|  | Endothelial Cell Dysfunction in Pulmonary Hypertension |
|  | Smooth Muscle Cell Dysfunction in Pulmonary Hypertension |
|  | BMP2 and Canonical WNT Signaling in Pulmonary Artery Endothelial Cells |
|  | Myocardial Remodeling |
|  | Proteins Involved in Myocardial Ischemia |
|  | Proteins Involved in Myocarditis |
|  | Contraction Due Vasospasm |
|  | Sebocyte Proliferation in Acne Vulgaris |
|  | Inflammatory Reaction in Acne Vulgaris |
|  | Keratinocyte Proliferation in Acne Vulgaris |
|  | Hair Follicle Keratinocyte Apoptosis |
|  | Atopic Dermatitis Onset |
|  | Mast-Cells Activation in Atopic Dermatitis |
|  | Proteins with Altered Expression in Melanoma |
|  | Cell Cycle Dysregulation in Melanoma |
|  | Melanoma |
|  | Apoptosis Block in Melanoma Cell |
|  | MITF as a Regulator of Melanoma Cell Development |
|  | IL17 Signaling in Psoriasis |
|  | Proteins Involved in Psoriasis |
|  | IL22 Induces Keratinocyte Proliferation in Psoriasis |
|  | Proteins with Altered Expression in Psoriasis |
|  | Th0-Cell Aberrant Activation in Systemic Lupus Erythematosus |
|  | Th1-Cell Function in Systemic Lupus Erythematosus |
|  | B-Cell Activation in Systemic Lupus Erythematosus |
|  | Th2-Cell Function in Systemic Lupus Erythematosus |
|  | Acute Cytotoxic CD8+T-Cell Response against Melanocytes |
|  | T-Cell Cytotoxic Response against Melanocytes in Vitiligo |
|  | Humoral Immunity in Vitiligo |
|  | CD8+ T-Cell Response to Self-Determinants in Diabetes |
|  | Treg-Cell Function in Diabetes Mellitus Type 1 |
|  | Natural Killer T-Cell Roles in Diabetes Mellitus Type 1 |
|  | non-Suppressive Treg-Cell in Diabetes Mellitus Type 1 |
|  | TLR2 Signaling in Treg-Cell in Type 1 Diabetes (Animal Model) |
|  | Natural Killer Cell in Diabetes Mellitus Type 1 |
|  | Toll-like Receptors in beta-Cell |
|  | Peripheral Tolerance to Autoantigens Recession in Diabetes Mellitus Type 1 |
|  | Treg-Cell Activation in Diabetes Mellitus |
|  | Donohue Syndrome Progression (Hypothesis) |
|  | Protein Kinase C in Diabetic Microangiopathy |
|  | Peripheral Tissue Microangiopathy |
|  | Tubular Cell Dysfunction in Progressive Diabetic Nephropathy |
|  | Endothelial Cell Dysfunction in Progressive Diabetic Nephropathy |
|  | Endothelial Cell Dysfunction in Early Diabetic Nephropathy |
|  | Proteins Involved in Diabetic Nephropathy |
|  | Mesangial Cell Dysfunction in Diabetic Nephropathy |
|  | Microangiopathy in Diabetic Neuropathy |
|  | Proliferative Diabetic Retinopathy |
|  | AGE Related Insulin Resistance in Skeletal Muscles |
|  | Adiponectin Synthesis Declines in Insulin Resistance |
|  | Hepatic Stellate Cells in non-Alcoholic Fatty Liver Disease |
|  | FOXO1 and SREBP-1C Role in beta-Cell Suppression (Rodent Model) |
|  | Hyperparathyroidism, Neonatal Severe |
|  | RET1 Mutation in Hyperparathyroidism |
|  | Hypothyrodism Associated Hyperprolactinemia |
|  | DRD2 Pharmacological Inhibition in Hyperprolactinemia |
|  | Ethanol Induced Efects in Hyperprolactinemia |
|  | GNRH Signaling Impairment in Hypogonadotrophic Hypogonadism |
|  | Myocyte Dysfunction in Hypoparathyroidism |
|  | Hypoparathyroidism, Primary |
|  | Thyroid Stimulating Hormone (TSH) Resistance in Congenital Hypothyroidism |
|  | Overt Hypothyroidism, Primary |
|  | Thyroid Stimulating Hormone (TSH) Secretion in Overt Hypothyroidism |
|  | Proteins Involved in Obesity |
|  | Prolactinoma Overiew |
|  | CD4+T-Cell Response in Celiac Disease |
|  | CD8+ T-Cell Response in Celiac Disease |
|  | Metastatic Colorectal Cancer |
|  | OipA Signaling in Helicobacter Pylori Infection |
|  | CagA Phosphorylation Dependent Signaling |
|  | EDN, NRG, NRTN, and GDNF/RET Signaling in Hirschsprung Disease |
|  | Th2-Cell Function in Ulcerative Colitis |
|  | T-Cell Cytotoxic Response in Ulcerative Colitis |
|  | B-Cell Acute Lymphoblastic Leukemia |
|  | B-Cell Chronic Lymphocytic Leukemia |
|  | Chronic Myeloid Leukemia |
|  | Follicular Lymphoma |
|  | Hodgkin Lymphoma |
|  | Osteoclast Activation in Multiple Myeloma |
|  | IL6/IGF1/VEGFA Signaling in Multiple Myeloma |
|  | Multiple Myeloma |
|  | T-Cell Acute Lymphoblastic Leukemia |
|  | Hepatocellular Carcinoma |
|  | Growth Factor Signaling in Hepatocellular Carcinoma |
|  | Proteins Involved in Pancreatic Cancer |
|  | Pancreatic Ductal Carcinoma |
|  | Growth Factor Signaling in Pancreatic Neoplasms |
|  | Pancreatic Neuroendocrine Tumors |
|  | TRPM7/8 in Pancreatic Adenocarcinoma |
|  | TGFBR Signaling in Pancreatic Neoplasms |
|  | Eosinophil Survival in Asthma |
|  | Il17 Signaling Related Neutrophilia in Asthma |
|  | Eosinophil Activation and Degranulation in Asthma |
|  | IL17F Signaling in Bronchial Epithelial Cell in Asthma |
|  | Airway Smooth Muscle Cell High Rate Proliferation |
|  | IgE Induces Airway Smooth Muscle Cell Proliferation |
|  | Neutrophil Degranulation via FPR1/IL8 |
|  | Antigen-Presenting Cell Role in Asthma |
|  | Th2-Cell Response in Asthma |
|  | Osteoclast Activation in Gout |
|  | TNF and IL1B Induce Metalloproteinase Synthesis in Osteoarthritis |
|  | IL1B Induced Arthralgia |
|  | Steroidogenesis Impairement in Polycystic Ovary Syndrome |
|  | Ovulation Block |
|  | Polycystic Ovary Syndrome |
|  | FSH Action in Polycystic Ovary Syndrome |
|  | Keratinocyte Activation in Psoriatic Arthritis |
|  | PTPN22 Role in Rheumatoid Arthritis |
|  | Synovial Fibroblast Proliferation in Rheumatoid Arthritis |
|  | Osteoclast Activation in Rheumatoid Arthritis |
|  | B-Cells Function in Systemic Scleroderma |
|  | Th2-Cells Function in Systemic Scleroderma |
|  | Proteins Involved in Systemic Scleroderma |
|  | Skin Fibrosis |
|  | WHIM Syndrome |
|  | Dentin Formation Disruption |
|  | CCR5 Signaling in Macrophages |
|  | CD8+ T-Cell Action Impairment in HIV Type 1 Infection |
|  | CD4+ T-Cell Function Decline in HIV |
|  | CD4+ T-Cell Death in HIV |
|  | Estrogen Effects on Pregnancy Gingivitis |
|  | IL1 Receptor Antagonist (IL1RN) Deficiency (DIRA) |
|  | Cowden Syndrome |
|  | Ca2+ Overload in Muscular Dystrophies |
|  | Muscular Dystrophies |
|  | IGF1 Role in Muscle Hypertrophy |
|  | Osteoblast Function Decline in Osteoporosis |
|  | Osteoclast Activation in Postmenopause |
|  | APP and Glutamate Signaling Cause Neuronal Dysfunction in Alzheimer Disease |
|  | BDNF Upregulation Triggered by Seizures |
|  | Apoptosis and Immediate Early Gene Activation in Epileptiform Disorders |
|  | Synaptic Exocytosis in Dementia |
|  | Glioblastoma, Primary |
|  | Glioblastoma, Secondary |
|  | Astrocytoma |
|  | Proteins Involved in Glioma |
|  | Growth Factor Signaling in Neuroblastoma |
|  | Neuroblastoma |
|  | ALK Associated Neuroblastoma |
|  | Proteins Involved in Breast Cancer Related to ESR1 Signaling Pathway |
|  | Proteins Involved in Breast Cancer Related to ERBB2/VEGFR/Akt Signaling Pathway |
|  | ERBB/VEGFR/Akt Signaling in Breast Cancer |
|  | IGF1R/AKT Signaling in Breast Cancer |
|  | ESR1/ERBB Positive Luminal Breast Cancer |
|  | Proteins Involved in Breast Cancer Related to IGF1R/Akt Signaling Pathway |
|  | Breast Cancer |
|  | ESR1 Signaling in Breast Cancer |
|  | Endometrial Cancer |
|  | Endometrioid Endometrial Cancer |
|  | Proteins Involved in Endometrial Cancer |
|  | Clear Cell Endometrial Cancer and Papillary Serous Endometrial Cancer |
|  | Angiogenesis in Endometriosis |
|  | Coagulation Factors and PAR1/2 Receptors (F2R/F2RL1) in Endometriosis |
|  | Leptin Role in Endometriosis |
|  | Proteins Involved in High-Grade Serous Ovarian Carcinoma |
|  | Endometrioid Ovarian Carcinoma |
|  | Proteins Involved in Endometrioid Ovarian Carcinoma |
|  | Clear Cell Ovarian Carcinoma |
|  | Proteins Involved in Mucinous Ovarian Carcinoma |
|  | EGFR and PTAFR Signaling in Ovarian Cancer |
|  | High-Grade Serous Ovarian Carcinoma |
|  | Low-Grade Serous Ovarian Carcinoma |
|  | Proteins Involved in Low-Grade Serous Ovarian Carcinoma |
|  | Ovarian Cancer |
|  | Proteins Involved in Clear Cell Ovarian Carcinoma |
|  | Mucinous Ovarian Carcinoma |
|  | MIF Role in Cholesteatoma |
|  | HMGB1/RAGE Signaling in Middle Ear Cholesteatoma |
|  | Subepithelial Fibroblasts in Middle Ear Cholesteatoma |
|  | Cholesteatoma |
|  | HPV E5 Regulates EDNRA and EGFR Signaling Pathway in Keratinocytes |
|  | Fatty Acid Synthase (FASN) Signaling |
|  | Androgen Receptor/FKBP5 Signaling |
|  | Prostate Cancer |
|  | TRPM8 Effects in Prostate Cancer (Hypothesis) |
|  | Proteins Involved in Prostate Cancer |
|  | Proteins Involved in Age-Related Macular Degeneration |
|  | Steroids Induced Cataract |
|  | VEGFA in Neovascular Glaucoma |
|  | Proteins Involved in Glaucoma |
|  | PGF2-alpha Effect on Treatment |
|  | Aminoglycosides and Cisplatin Induced Ototoxicity (Mouse Model) |
|  | Cytokines Trigger Otitis Media |
|  | MUC5AC Expression by NTHi and S. Pneumoniae |
|  | Alveolar Macrophage Negative Regulation Declined in COPD |
|  | VEGFA Low Expression Level in Chronic Bronchitis |
|  | VEGFA High Expression Level in Pulmonary Emphysema |
|  | Alveolar Epithelial Cell Dysfunction in COPD |
|  | Polycystins Mutation Effects in Cystic Kidney Disease |
|  | Proteins Involved in Erectile Dysfunction |
|  | Plasmin Role in Glomerulonephritis |
|  | Endothelial Cell Dysfunction in Glomerulonephritis |
|  | Mesangial Cell Dysfunction in Glomerulonephritis |
|  | Interstitial Fibroblasts in Pyelonephritis |
|  | Sertoli Cells Dysfunction Causes Male Infertility |
|  | Proteins Involved in Male Infertility |
|  | CD38/CD3 -> JUN/FOS/NF-kB Signaling in T-cell Proliferation |
|  | CD38/CD19 -> JUN/FOS/NF-kB Signaling in B-cell Proliferation |
|  | CD38 -> cADPR/Calcium Signaling |
|  | CD157 -> cADPR/Calcium Signaling |
|  | S100P Expression Target |
|  | HMGB1 Expression Targets |
|  | TTR Expression Targets |
|  | S100A Expression Targets |
|  | S100B Expression Targets |
|  | ANGPT1 -> CREB/CREBBP Expression Targets |
|  | ANGPT2 -> AP-1/CREBBP/MYC Expression Targets |
|  | AREG -> CREB/CREBBP Expression Targets |
|  | AREG -> AP-1 Expression Targets |
|  | Noradrenaline/Gs Expression Targets |
|  | Dopamine/Gs Expression Targets |
|  | Serotonin/Gs Expression Targets |
|  | Noreadrenaline/Gq Expression Targets |
|  | Epinephrine/Gs Expression Targets |
|  | Acetylcholine Expression Targets |
|  | Serotonin/Gq Expression Targets |
|  | Epinephrine/Gq Expression Targets |
|  | Dopamine/Gi Expression Targets |
|  | B-Cell Receptor -> AP-1 Signaling |
|  | BTC -> AP-1/ATF/CREB Expression Targets |
|  | BTC -> EP300/ETS/ETV/SP1 Expression Targets |
|  | GLP1R -> ARRB1 Signaling |
|  | IGF1R -> ARRB1/ERK1/3 Signaling |
|  | AGTR1 -> ARRB1/ARRB2 Signaling |
|  | SELE -> ELK-SRF Signaling |
|  | CD19 Expression Targets |
|  | CD81 -> AP-1 Expression Targets |
|  | CD247 Expression Targets |
|  | gamma Globulins Expression Targets |
|  | FCGR3A Expression Targets |
|  | CD19/CR2 Expression Targets |
|  | CD72 -> CREB/CREBBP Expression Targets |
|  | CD8 -> ATF/CREB/CREBBP Expression Targets |
|  | CD80 -> NF-kB Expression Targets |
|  | CD86 -> NF-kB Expression Targets |
|  | CXCL6 Expression Targets |
|  | CXCL1 Expression Targets |
|  | CXCL2 Expression Targets |
|  | CXCL3 Expression Targets |
|  | CXCL5 Expression Targets |
|  | CHRNA7 -> CREB Signaling |
|  | Clozapine Induced Granulocytopenia |
|  | EGF -> AP-1/ATF Expression Targets |
|  | EGF -> CREB/CREBBP/ELK/SRF/MYC Expression Targets |
|  | EGF -> TP53 Expression Targets |
|  | EGFR/ERBB2 -> TP53 Signaling |
|  | ERBB2/3 -> EP300/ETS/ETV/SP1 Signaling |
|  | EGFR -> AP-1/ATF2 Signaling |
|  | EGFR -> AP-1/CREB/ELK/SRF/MYC Signaling |
|  | EREG -> CREB Expression Target |
|  | EREG -> EP300/SP1 Expression Targets |
|  | EREG -> AP-1/ATF Expression Targets |
|  | Erythropoietin -> AP-1/MYC/CREB Expression Targets |
|  | Erythropoietin -> ELK/SRF Expression Targets |
|  | PECAM1 Expression Targets |
|  | NCAM1 Expression Targets |
|  | F2 -> AP-1/CREB/ELK/SRF/SP1 Expression Targets |
|  | PLG -> AP-1/CREB/ELK/SRF/SP1 Expression Targets |
|  | FGF9 -> AP-1/CREB/MYC Expression Targets |
|  | FGF10 -> AP-1/CREB/CREBBP/MYC Expression Targets |
|  | FGF1 -> AP-1/CREB/ELK/SRF/MYC Expression Targets |
|  | FGF7 -> RUNX Expression Targets |
|  | FGF8 -> AP-1/CREB/MYC Expression Targets |
|  | FGF1 -> RUNX Expression Targets |
|  | FGF18 -> AP-1/CREB Expression Targets |
|  | FGF8 -> RUNX Expression Targets |
|  | FGF9 -> RUNX Expression Targets |
|  | FGF4 -> AP-1/MYC Expression Targets |
|  | FGF7 -> AP-1/CREB/CREBBP/MYC Expression Targets |
|  | FGF2 -> RUNX Expression Targets |
|  | FGF2 -> AP-1/CREB/CREBBP/ELK/SRF/MYC Expression Targets |
|  | FGFR -> RUNX2 Signaling |
|  | FGFR -> AP-1/CREB/CREBBP/ELK/SRF/MYC Signaling |
|  | GCG Expression Targets |
|  | ADCYAP1 Expression Targets |
|  | FSHR Expression Targets |
|  | Glutamate/Gq Expression Targets |
|  | VIP Expression Targets |
|  | GH1/GHR -> NF-kB/ELK/SRF/MYC Expression Targets |
|  | CSH1/GHR Expression Targets |
|  | HBEGF -> AP-1/ATF Expression Targets |
|  | HBEGF -> TP53 Expression Targets |
|  | HBEGF -> EP300/ETS/ETV/SP1 Expression Targets |
|  | HBEGF -> CREB/MYC Expression Targets |
|  | HGF -> AP-1/CREB/ELK/SRF/MYC Expression Targets |
|  | HGFR -> AP-1/CREB/MYC Signaling |
|  | TAM Receptor Signaling Activation in Cancer |
|  | RAS/RAF/MAPK Signaling Activation in Cancer |
|  | RAS/RAF/MAPK Signaling Activation by Blocking of Tumor Suppressors |
|  | TGFB Signaling activation by Blocking of Tumor Suppressors |
|  | Oxidized LDL/OLR1 Activates Cell Proliferation in Cancer |
|  | HIF1A in Vasculogenic Mimicry of Cancer |
|  | VEGFA Dependent Angiogenesis in Cancer |
|  | ANGPT/TEK Stimulates Endothelial Cell Migration in Cancer |
|  | VEGF Independent Angiogenesis in Cancer |
|  | Integrins in Cancer Cell Motility, Invasion and Survival |
|  | Proteins with Altered Expression in Cancer Metastases |
|  | BSG (CD147) in Cancer Cells Motility, Invasion and Survival |
|  | MTOR/TORC in Cancer Cell Motility and Invasion |
|  | Hyaluronic Acid, CD44 and HMMR in Cancer Cell Invasion and Survival |
|  | CDH2 Activation Promotes Cancer Cell Migration and Survival |
|  | TGFB Family in Epithelial to Mesenchymal Transition in Cancer |
|  | Lactate as a Signaling Molecule in Cancer Cells |
|  | Treg Cells Promote Immunosuppression in Cancer Immune Escape |
|  | HCAR1/HCAR2 -> MAPK Signaling |
|  | IGF2 -> HIF1A/MYC Expression Targets |
|  | IGF1 -> ELK/SRF/HIF1A/MYC/SREBF Expression Targets |
|  | Insulin -> ELK/SRF/HIF1A/MYC/SREBF Expression Targets |
|  | AGER -> CREB/SP1 Signaling |
|  | NCAM1 -> CREB/ELK/SRF/MYC Signaling |
|  | CD19 -> JUN/ELK Signaling |
|  | PECAM -> SP1 Signaling |
|  | FcIgER -> ELK-SRF Signaling |
|  | ICAM1 -> AP-1/CREB/ELK/SRF Signaling |
|  | IGF1R -> ELK/SRF/HIF1A/MYC/SREBF Signaling |
|  | InsulinR -> ELK/SRF/SREBF Signaling |
|  | FibronectinR -> AP-1/ELK/SRF/SREBF Signaling |
|  | Fibronectin Expression Targets |
|  | CTGF -> AP-1/CREB/MYC Expression Targets |
|  | ICAM1 Expression Targets |
|  | IL6R -> CEBP/ELK/SRF Signaling |
|  | IL2R -> ELK/SRF/MYC Signaling |
|  | IL1R -> NF-kB Signaling |
|  | IL4R -> ELK/SRF/HMGY Signaling |
|  | IL16 -> AP-1 Expression Targets |
|  | IL6 Expression Targets |
|  | IL4 Expression Targets |
|  | IL2 Expression Targets |
|  | UrokinaseR -> ELK/SRF Signaling |
|  | PTPRC -> BCL6 Expression Targets |
|  | Leptin -> ELK/SRF Expression Targets |
|  | Prostaglandin F Expression Targets |
|  | Thromboxane A2 Expression Targets |
|  | LPA Expression Targets |
|  | PAF/Gq -> AP-1/ATF1/CREB/ERK/SRF Expression Targets |
|  | Dronabinol/Anandamide Expression Targets |
|  | Morphine Expression Targets |
|  | S1P Expression Targets |
|  | PGE1 Expression Targets |
|  | MTNR1 Signaling |
|  | NRG1 -> EP300/ETS/ETV/SP1 Expression Targets |
|  | NRG1 -> CREB/CREBBP/ELK/SRF/MYC Expression Targets |
|  | NRG1 -> TP53 Expression Target |
|  | NRG1 -> AP-1/ATF Expression Targets |
|  | NGF -> AP-1/TP53/MYC Expression Targets |
|  | NGF -> FOXO/MYCN/ELK-SRF Expression Targets |
|  | BDNF Expression Targets |
|  | NTF3 Expression Targets |
|  | NTF4 Expression Targets |
|  | NGF -> CREB/CEBPB/MEF2A Expression Targets |
|  | NOTCH -> TCF3 Signaling |
|  | NOTCH Expression Targets |
|  | Adenosine Expression Targets |
|  | OR1A1 -> GNAS Signaling |
|  | PDGFB -> AP-1/CREB/MYC Expression Target |
|  | PDGFC -> CREB Expression Target |
|  | PDGFD -> AP-1 Expression Targets |
|  | PDGF -> AP-1/CREB/CREBBP/MYC Expression Targets |
|  | PDGFR -> AP-1/MYC Signaling |
|  | AVP/Gs -> CREB/ELK/SRF/AP-1/EGR Expression Targets |
|  | GNRH1 Expression Targets |
|  | NTS Expression Targets |
|  | GAST Expression Targets in GAST Expression Targets |
|  | AGT -> TP53 Expression Targets |
|  | GNRH2 Expression Targets |
|  | OXT Expression Targets |
|  | POMC Expression Targets |
|  | CCL11 Expression Targets |
|  | EDN1 Expression Targets |
|  | AGT -> CREB Expression Targets |
|  | NPY Expression Targets |
|  | IFNA1/Gq Expression Targets |
|  | AGT -> ELK/SRF Expression Targets |
|  | TAC1 Expression Targets |
|  | EDN3 Expression Targets |
|  | AVP/Gq -> CREB/ELK/SRF/AP-1/EGR Expression Targets |
|  | PRL/GHR -> NF/kB/ELK/SRF/MYC Expression Targets |
|  | PDCD1 -> AP-1 Expression Targets |
|  | PTPRC -> BCL6 Signaling |
|  | AngiopoietinR -> AP-1 Signaling |
|  | GDNF -> HSF1 Signaling |
|  | EphrinB -> MAPK/JUN/FOS Signaling |
|  | NTRK -> AP-1/CREB/ELK/SRF/MYC/SMAD3/TP53 Signaling |
|  | GAS6 -> AP-1/CREB Expression Targets |
|  | FIGF -> AP-1 Expression Target |
|  | KITLG -> AP-1/CREB/CREBBP/MYC Expression Targets |
|  | GDNF -> HSF1 Expression Targets |
|  | CSF1 -> AP-1/CREB/CREBBP/MYC Expression Targets |
|  | PGF -> AP-1/CREB/CREBBP/MYC Expression Targets |
|  | FLT3LG -> AP-1/CREB/CREBBP Expression Targets |
|  | Lysophosphatidic Acid/LPARs Signaling |
|  | Ras-GRF Regulation Signaling |
|  | Androgen Receptor non-Genomic Signaling |
|  | FOXO1 Signaling |
|  | MERTK Signaling |
|  | TGF-beta Signaling |
|  | AXL Receptor Tyrosine Kinase Signaling |
|  | VEGF Signaling |
|  | Estrogens/ESR1 non-Genomic Signaling |
|  | EGFR Signaling |
|  | ERK/MAPK Canonical Signaling |
|  | TCR -> AP-1 Expression Targets |
|  | T-Cell Receptor -> AP-1 Signaling |
|  | TGFA -> TP53 Expression Targets |
|  | TGFA/AP-1/ATF Expression Targets |
|  | TGFA -> CREB/CREBBP/ELK-SRF/MYC Expression Targets |
|  | FASLG Expression Targets |
|  | TNFSF10 Expression Targets |
|  | LTA Expression Targets |
|  | TNFSF14 Expression Targets |
|  | TNF -> AP-1 Expression Targets |
|  | CD72 -> AP-1 Expression Targets |
|  | CD40LG -> NF-kB/ELK/SRF -> CREB/NFATC Expression Targets |
|  | TNFSF13 Expression Targets |
|  | TNFSF13B Expression Targets |
|  | TNF -> ELK-SRF Expression Targets |
|  | TNF -> CREB Expression Targets |
|  | TNF -> TP53/ATF Expression Targets |
|  | EDA Expression Targets |
|  | EctodysplasinR -> AP-1 Signaling |
|  | TNFR -> CREB/ELK-SRF Signaling |
|  | TNFR -> AP-1/ATF/TP53 Signaling |
|  | NGFR -> AP-1/CEBPB/CREB/ELK/SRF/TP53 Signaling |
|  | TNFRSF6 -> HSF1 Signaling |
|  | TNFRSF1A -> AP-1/ATF/TP53 Signaling |
|  | TNFRSF1A -> CREB/ELK-SRF Signaling |
|  | Thrombopoietin -> AP-1/CREB/CREBBP/MYC Expression Targets |
|  | Thrombopoietin -> SP1 Expression Targets |
|  | IL1B -> NO Expression Targets |
|  | IL1B -> PGE2 Expression Targets |
|  | IL1B Expression Targets |
|  | IL1A Expression Targets |
|  | PLAU -> ELK-SRF/AP-1 Expression Targets |
|  | VEGFA -> ATF/CREB/ELK-SRF Expression Targets |
|  | VEGFC -> ATF Expression Target |
|  | VEGFA -> AP-1/CREBBP/MYC Expression Targets |
|  | VEGFR -> AP-1/CREB/MYC Signaling |
|  | VEGFR -> ATF/CREB/ELK-SRF Signaling |
|  | pathway 3 |
|  | ace2 |
|  | prarg negative regulators, ps-positive |
|  | pprarg neg, uknown targets, ps-positive |
|  | PPARG negative regulators |
|  | pparg expr target\_regulators |
|  | Neighbors of disease exacerbation |
|  | Neighbors of tumor progression |
|  | Neighbors of cancer progression |
|  | Neighbors of tumor size |
|  | Neighbors of infarct size |
|  | Neighbors of functional recovery |
|  | Neighbors of insulin sensitivity |
|  | Neighbors of clinical stage |
|  | Neighbors of lethality |
|  | Neighbors of tumor regression |
|  | Neighbors of disease-free survival |
|  | Neighbors of macrophage count |
|  | Neighbors of bone mass |
|  | Neighbors of tumor promotion |
|  | Neighbors of neutrophil count |
|  | Neighbors of bone mineral density |
|  | Neighbors of radioresistance |
|  | Neighbors of cell density |
|  | Neighbors of progression-free survival |
|  | Neighbors of apoptosis rate |
|  | Neighbors of shear stress |
|  | Neighbors of viral load |
|  | Neighbors of systolic blood pressure |
|  | Neighbors of blood glucose |
|  | Neighbors of energy expenditure |
|  | Neighbors of vascular tone |
|  | Neighbors of radiosensitivity |
|  | Neighbors of left ventricular ejection fraction |
|  | Neighbors of cell permeability |
|  | Neighbors of fertility |
|  | Neighbors of recurrence rate |
|  | Neighbors of lymphocyte count |
|  | Neighbors of skin thickness |
|  | Neighbors of metabolic response |
|  | Neighbors of disease exacerbation |
|  | Neighbors of tumor progression |
|  | Neighbors of cancer progression |
|  | Neighbors of tumor size |
|  | Neighbors of infarct size |
|  | Neighbors of functional recovery |
|  | Neighbors of insulin sensitivity |
|  | Neighbors of clinical stage |
|  | Neighbors of lethality |
|  | Neighbors of tumor regression |
|  | Neighbors of disease-free survival |
|  | Neighbors of macrophage count |
|  | Neighbors of bone mass |
|  | Neighbors of tumor promotion |
|  | Neighbors of neutrophil count |
|  | Neighbors of bone mineral density |
|  | Neighbors of radioresistance |
|  | Neighbors of cell density |
|  | Neighbors of progression-free survival |
|  | Neighbors of apoptosis rate |
|  | Neighbors of shear stress |
|  | Neighbors of viral load |
|  | Neighbors of systolic blood pressure |
|  | Neighbors of blood glucose |
|  | Neighbors of energy expenditure |
|  | Neighbors of vascular tone |
|  | Neighbors of radiosensitivity |
|  | Neighbors of left ventricular ejection fraction |
|  | Neighbors of cell permeability |
|  | Neighbors of fertility |
|  | Neighbors of recurrence rate |
|  | Neighbors of lymphocyte count |
|  | Neighbors of skin thickness |
|  | Neighbors of metabolic response |
|  | Neighbors of cell development |
|  | Neighbors of immune response |
|  | Neighbors of T-cell development |
|  | Neighbors of inflammatory response |
|  | Neighbors of wound healing |
|  | Neighbors of angiogenesis |
|  | Neighbors of innate immune response |
|  | Neighbors of cell function |
|  | Neighbors of macrophage activation |
|  | Neighbors of cell formation |
|  | Neighbors of adaptive immune response |
|  | Neighbors of osteoclast development |
|  | Neighbors of epithelial to mesenchymal transition |
|  | Neighbors of immunity |
|  | Neighbors of cell proliferative response |
|  | Neighbors of chemotaxis |
|  | Neighbors of dendritic cell differentiation |
|  | Neighbors of cell survival |
|  | Neighbors of osteoclast differentiation |
|  | Neighbors of cell phenotype |
|  | Neighbors of T-cell proliferation |
|  | Neighbors of cell adhesion |
|  | Neighbors of T-cell activation |
|  | Neighbors of endothelial cell proliferation |
|  | Neighbors of fibrogenesis |
|  | Neighbors of tumor growth |
|  | Neighbors of dendritic cell development |
|  | Neighbors of cell count |
|  | Neighbors of cell infiltration |
|  | Neighbors of cell interaction |
|  | Neighbors of SMC proliferation |
|  | Neighbors of lumen formation |
|  | Neighbors of endothelial cell migration |
|  | Neighbors of adipogenesis |
|  | Neighbors of lipid storage |
|  | Neighbors of cell damage |
|  | Neighbors of pregnancy |
|  | Neighbors of aging |
|  | Neighbors of cell motility |
|  | Neighbors of bone resorption |
|  | Neighbors of monocyte migration |
|  | Neighbors of fibroblast proliferation |
|  | Neighbors of adipocyte differentiation |
|  | Neighbors of microglial activation |
|  | Neighbors of neutrophil recruitment |
|  | Neighbors of regeneration |
|  | Neighbors of cell migration |
|  | Neighbors of cancer growth |
|  | Neighbors of stem cell proliferation |
|  | Neighbors of smooth muscle cell migration |
|  | Neighbors of hepatic regeneration |
|  | Neighbors of transendothelial migration |
|  | Neighbors of phagocytosis |
|  | Neighbors of osteoclast formation |
|  | Neighbors of cell invasion |
|  | Neighbors of heart function |
|  | Neighbors of hemopoiesis |
|  | Neighbors of ossification |
|  | Neighbors of autophagy |
|  | Neighbors of monocyte adhesion |
|  | Neighbors of transcription activation |
|  | Neighbors of senescence |
|  | Neighbors of hepatocyte proliferation |
|  | Neighbors of neuroprotection |
|  | Neighbors of stem cell differentiation |
|  | Neighbors of endothelial cell function |
|  | Neighbors of neuronal death |
|  | Neighbors of cell growth |
|  | Neighbors of ROS generation |
|  | Neighbors of epithelial cell proliferation |
|  | Neighbors of first trimester pregnancy |
|  | Neighbors of cancer cell growth |
|  | Neighbors of sensitization |
|  | Neighbors of cellular immune response |
|  | Neighbors of cellular senescence |
|  | Neighbors of colony formation |
|  | Neighbors of cell differentiation |
|  | Neighbors of keratinocyte proliferation |
|  | Neighbors of stem cell migration |
|  | Neighbors of cell homeostasis |
|  | Neighbors of psoriasis |
|  | Neighbors of inflammatory disease |
|  | Neighbors of rheumatoid arthritis |
|  | Neighbors of atherosclerosis |
|  | Neighbors of fibrosis |
|  | Neighbors of injury |
|  | Neighbors of atherogenesis |
|  | Neighbors of vascular remodeling |
|  | Neighbors of metastasis |
|  | Neighbors of carcinogenesis |
|  | Neighbors of inflammation |
|  | Neighbors of pathological angiogenesis |
|  | Neighbors of obesity |
|  | Neighbors of vascular disease |
|  | Neighbors of diabetes mellitus |
|  | Neighbors of asthma |
|  | Neighbors of pneumonia |
|  | Neighbors of hyperplasia |
|  | Neighbors of osteoarthritis |
|  | Neighbors of liver fibrosis |
|  | Neighbors of reperfusion injury |
|  | Neighbors of airway inflammation |
|  | Neighbors of hepatitis |
|  | Neighbors of insulin resistance |
|  | Neighbors of infection |
|  | Neighbors of renal injury |
|  | Neighbors of preeclampsia |
|  | Neighbors of macrophage infiltration |
|  | Neighbors of neuroinflammation |
|  | Neighbors of death |
|  | Neighbors of type 1 diabetes |
|  | Neighbors of ischemic stroke |
|  | Neighbors of heart disease |
|  | Neighbors of diabetic nephropathy |
|  | Neighbors of systemic sclerosis |
|  | Neighbors of hepatocellular carcinoma |
|  | Neighbors of autoimmunity |
|  | Neighbors of acute lung injury |
|  | Neighbors of colorectal cancer |
|  | Neighbors of breast cancer |
|  | Neighbors of ischemia |
|  | Neighbors of melanoma |
|  | Neighbors of interstitial fibrosis |
|  | Neighbors of neoplasm invasion |
|  | Neighbors of kidney disease |
|  | Neighbors of stroke |
|  | Neighbors of cancer |
|  | Neighbors of cerebral ischemia |
|  | Neighbors of type 2 diabetes |
|  | Neighbors of neoplasm |
|  | Neighbors of atrophy |
|  | Neighbors of diet-induced obesity |
|  | Neighbors of respiratory hypersensitivity |
|  | Neighbors of myocardial fibrosis |
|  | Neighbors of rupture |
|  | Neighbors of endothelial cell dysfunction |
|  | Neighbors of liver disease |
|  | Neighbors of prostate cancer |
|  | Neighbors of hypertrophy |
|  | Neighbors of hypertension |
|  | Neighbors of intimal hyperplasia |
|  | Neighbors of proteinuria |
|  | Neighbors of monocyte |
|  | Neighbors of macrophage |
|  | Neighbors of pneumocyte |
|  | Neighbors of endothelial cell |
|  | Neighbors of granulocyte |
|  | Neighbors of stem cell |
|  | Neighbors of chondrocyte |
|  | Neighbors of heart |
|  | Neighbors of cerebral cortex |
|  | Neighbors of nervous system |
|  | Neighbors of trachea |
|  | Neighbors of PKC |
|  | Neighbors of mitogen-activated protein kinase |
|  | Neighbors of interferon |
|  | Neighbors of PDGF-BB |
|  | Neighbors of heat shock protein 90 |
|  | Neighbors of vitronectin receptor (integrin) |
|  | sars-cov no binding |
|  | 1\_Inflammation in keratinocytes in the pathology of acne |
|  | 2\_Hyperseborrhea in acne vulgaris |
|  | 2\_Acute phase initiation in atopic dermatitis |
|  | 2\_Interleukin-17 and interleukin-22 signaling in psoriasis |
|  | 1\_The onset of atopic dermatitis |
|  | 1\_TNF and IL1B provoke ECM degradation in osteoarthritis |
|  | 2\_Osteoblast-mediated bone demineralization |
|  | 1\_Formation of cysts in polycystic kidney disease |
|  | 3\_The role of interstitial fibroblasts in pyelonephritis |
|  | 3\_Enhanced angiogenesis in endometriosis |
|  | 2\_Impaired steroidogenesis in PCOS |
|  | 1\_Disruption of luteinizing hormone and follicle-stimulating hormone secretion in PCOS |
|  | 2\_Pathogens stimulate mucins expression in the middle ear |
|  | 2\_Oxidized LDL causes endothelial dysfunction in atherosclerosis |
|  | 2\_Cardiomyocyte hypertrophy |
|  | 3\_Dysfunction of smooth muscle cells in hypertension |
|  | 1\_Rise in aldosterone synthesis in adrenal zona glomerulosa cells |
|  | 2\_The intense proliferation and contractile activity of smooth muscle cells in pulmonary hypertension |
|  | 1\_Cigarette smoke causes dysfunction and production of inflammatory mediators in alveolar epithelial cells |
|  | 3\_Role of alveolar macrophages in pulmonary tissue destruction in COPD |
|  | 1\_Th2 cell response in asthma |
|  | 3\_1\_Mucus accumulation in asthma |
|  | 2\_2\_Eosinophilia and neutrophilia in asthma: eosinophil apoptosis |
|  | 4\_Mucin hyperproduction in goblet and mucous cells in COPD |
|  | 2\_CD8+ T-cell response and mucosal damage in celiac disease |
|  | 1\_1\_Insulin synthesis and secretion: insulin processing and storage |
|  | 4\_1\_Insulin resistance: insulin signaling |
|  | 3\_Islet beta-cell destruction in T1D |
|  | 1\_Microangiopathy in diabetic neuropathy |
|  | 2\_1\_General pathways of thyroid hormone signaling |
|  | public list |
|  | New Pathway |
|  | HRAS->RAF->MAPK1/3 |
|  | KRAS->RAFs->MAPK1/3 |
|  | NRAS->RAFs->MAPK1/3 |
|  | Other RASs-RAFs-MAPK1/3 |
|  | mapk1 |
|  | New Pathway |
|  | mapk1 protein modificators |
|  | Interleukin-17 and interleukin-22 signaling in psoriasis |

---

|  |  |
| --- | --- |
| Group | Proteins Involved in Cowden Syndrome |
|  | other partners for SARS |

---

|  |  |
| --- | --- |
| Source | GO component |
|  | ResNet |
|  | curated |

---

|  |  |
| --- | --- |
| MedScan ID | 5595 |

---

|  |  |
| --- | --- |
| LocusLink ID | 5595 |
|  | 26417 |
|  | 50689 |
|  | 327402 |
|  | 531391 |
|  | 19095 |
|  | 399480 |
|  | 557882 |
|  | 326417 |
|  | 107837 |
|  | 17757 |

---

|  |  |
| --- | --- |
| Alias | mitogen activated protein kinase ERK 1 |
|  | OTTMUSP00000017737 |
|  | OTTHUMP00000174541 |
|  | ERK1 protein kinase |
|  | OTTMUSP00000017736 |
|  | OTTHUMP00000174538 |
|  | p44 MAP kinase |
|  | mitogen-activated 3 |
|  | protein kinase, mitogen activated 3 (extracellular-signal-regulated kinase 1) |
|  | Esrk1 |
|  | ERT2 |
|  | Extracellular signal-regulated kinase 1 |
|  | MAPK p44 |
|  | MAP kinase ERK1 |
|  | protein kinase, mitogen-activated III |
|  | extracellular signal-related kinase 1c |
|  | rMNK1 |
|  | ERK1 |
|  | p44erk1 |
|  | Insulin-stimulated MAP2 kinase |
|  | pp42/MAP kinase |
|  | ERK1 MAP kinase |
|  | OTTHUMP00000174540 |
|  | MAP kinase III |
|  | HUMKER1A |
|  | Mtap2k |
|  | HS44KDAP |
|  | Extracellular signal-regulated kinase I |
|  | PRKM3 |
|  | p44/42 mapk |
|  | mitogen activated protein kinase p44 |
|  | mitogen-activated 3 (MAP kinase 3 |
|  | mitogen activated kinase 3 |
|  | extracellular signal-related kinase 1 |
|  | mitogen-activated III |
|  | MGC20180 |
|  | MAP kinase isoform p44 |
|  | mitogen activated protein kinase III |
|  | p44 ERK |
|  | Microtubule-associated protein-2 kinase |
|  | MAP kinase 3 |
|  | Esrk I |
|  | extracellular signal-regulated kinase (ERK) 1 |
|  | MAPK3 |
|  | protein kinase, mitogen-activated 3 (MAP kinase 3, p44) |
|  | mitogen-activated 3 (map kinase 3; p44) |
|  | p44 mitogen activated protein kinase |
|  | extracellular signaling-regulated kinase 1 |
|  | protein kinase, mitogen-activated 3 |
|  | OTTMUSP00000017735 |
|  | p44erk I |
|  | ERK I |
|  | protein kinase ERK1 |
|  | p44mapk |
|  | ERK-1 |
|  | p44-ERK1 |
|  | p44-MAPK |
|  | MAPK 1 |
|  | microtubule-associated protein 2 kinase |
|  | mitogen-activated protein kinase 3 |
|  | p44 |
|  | Mnk1 |
|  | MAPK1 |
|  | MAP kinase 1 |
|  | MAPK 3 |
|  | mitogen-activated protein kinase 1 |
|  | p44) |
|  | mitogen activated protein kinase 3 |
|  | protein kinase, mitogen activated kinase 3 |
|  | extracellular-signal-regulated kinase 1 |
|  | Protein kinase mitogen activated 3 (extracellular-signal-regulated kinase 1 ERK1) |
|  | protein kinase, mitogen activated 3 (extracellular-signal-regulated kinase 1, ERK1) |
|  | LOC557882 |
|  | similar to glycogen synthase kinase 3 |
|  | scaffold |
|  | wu:fi06b09 |
|  | chromosome |
|  | Serum phospholipid level QTL 1 |
|  | similar to extracellular signal-regulated kinase I |
|  | similar to microtubule-associated protein-2 kinase |
|  | similar to extracellular signal-regulated kinase 1 |
|  | fi06b09 |
|  | Lil2 |
|  | Serum phospholipid level QTL I |
|  | Lipid Level QTL 2 |
|  | Lipid Level QTL II |
|  | protein kinase |
|  | kinase, mitogen-activated 3 (MAP kinase 3; p44) |
|  | Scl23 |
|  | protein kinase, mitogen-activated 3 (MAP kinase 3; p44) |
|  | similar to extracellular signal-related kinase 1c |

---

|  |  |
| --- | --- |
| EC Number | 2.7.11.24 |
|  | 2.7.11.1 |
|  | 2.7.1.37 |
|  | 2.7.1.- |

---

|  |  |
| --- | --- |
| GO ID | 0005524 |
|  | 0004707 |
|  | 0004708 |
|  | 0019902 |
|  | 0001784 |
|  | 0097110 |
|  | 0030509 |
|  | 0060020 |
|  | 0006975 |
|  | 0070371 |
|  | 0038095 |
|  | 0038096 |
|  | 0000165 |
|  | 0000187 |
|  | 0000186 |
|  | 0007568 |
|  | 0006915 |
|  | 0019369 |
|  | 0007411 |
|  | 0061308 |
|  | 0051216 |
|  | 0072584 |
|  | 0007049 |
|  | 0034198 |
|  | 0071276 |
|  | 1903351 |
|  | 0071260 |
|  | 0071310 |
|  | 0034614 |
|  | 0071356 |
|  | 0046697 |
|  | 0060324 |
|  | 0008543 |
|  | 0070498 |
|  | 0035556 |
|  | 0031663 |
|  | 0060425 |
|  | 2000657 |
|  | 0042473 |
|  | 0018105 |
|  | 0038083 |
|  | 0016310 |
|  | 0030168 |
|  | 0070374 |
|  | 0031281 |
|  | 0002741 |
|  | 0010628 |
|  | 0035066 |
|  | 0033129 |
|  | 0010759 |
|  | 1905050 |
|  | 0001934 |
|  | 0051973 |
|  | 1904355 |
|  | 0032212 |
|  | 0045944 |
|  | 0045727 |
|  | 1904417 |
|  | 0006468 |
|  | 0065003 |
|  | 0051090 |
|  | 0090170 |
|  | 0030641 |
|  | 1900034 |
|  | 0051493 |
|  | 2000641 |
|  | 0010468 |
|  | 0030278 |
|  | 0014066 |
|  | 0032872 |
|  | 0070849 |
|  | 0043330 |
|  | 0009636 |
|  | 0019233 |
|  | 0051403 |
|  | 0048538 |
|  | 0030878 |
|  | 0060440 |
|  | 0006361 |
|  | 0016032 |
|  | 0005794 |
|  | 0005901 |
|  | 0005737 |
|  | 0005856 |
|  | 0005829 |
|  | 0005769 |
|  | 0005925 |
|  | 0005770 |
|  | 0005739 |
|  | 0005635 |
|  | 0005654 |
|  | 0005634 |
|  | 0005886 |
|  | 0032991 |
|  | 0031143 |
|  | 0016301 |
|  | 0000166 |
|  | 0004672 |
|  | 0004674 |
|  | 0016740 |
|  | 0009887 |
|  | 0006974 |
|  | 0014032 |
|  | 0032496 |
|  | 0007165 |
|  | 0006351 |
|  | 0016020 |
|  | 0060397 |
|  | 0000189 |
|  | 0006461 |
|  | 0070062 |
|  | 0015630 |
|  | 0043234 |
|  | 0045893 |
|  | 0002755 |
|  | 0002756 |
|  | 0007265 |
|  | 0035666 |
|  | 0007596 |
|  | 0034605 |
|  | 0019221 |
|  | 0007173 |
|  | 0010467 |
|  | 0045087 |
|  | 0008286 |
|  | 0048011 |
|  | 0007264 |
|  | 0034166 |
|  | 0034134 |
|  | 0034138 |
|  | 0034142 |
|  | 0034146 |
|  | 0034162 |
|  | 0038123 |
|  | 0038124 |
|  | 0002224 |
|  | 0006360 |
|  | 0048010 |
|  | 0016772 |
|  | 0019048 |
|  | 0005730 |
|  | 0008063 |
|  | 0034130 |
|  | 0008152 |
|  | 0023014 |
|  | 0005622 |
|  | 0007243 |
|  | 0044419 |
|  | 0030528 |
|  | 0005626 |
|  | 0005625 |
|  | 0023034 |
|  | 0045941 |
|  | 0005515 |
|  | 0005575 |
|  | 0003700 |
|  | 0007242 |
|  | 0004682 |
|  | 0008372 |
|  | 0004713 |
|  | 0004691 |
|  | 0000074 |

---

|  |  |
| --- | --- |
| KEGG ID | hsa:5595 |
|  | mmu:26417 |
|  | rno:50689 |

---

|  |  |
| --- | --- |
| MedLine Reference | 12783880 |
|  | 17161379 |
|  | 15314156 |
|  | 16616413 |
|  | 12270934 |
|  | 16368546 |
|  | 17581212 |
|  | 15247297 |
|  | 15778391 |
|  | 17164422 |
|  | 12218048 |
|  | 17615382 |
|  | 12218045 |
|  | 12971962 |
|  | 10780709 |
|  | 8424957 |
|  | 16871540 |
|  | 11994544 |
|  | 12408764 |
|  | 12200131 |
|  | 9325171 |
|  | 15671028 |
|  | 16343798 |
|  | 8806671 |
|  | 11579140 |
|  | 16873684 |
|  | 1889806 |
|  | 12805214 |
|  | 12792650 |
|  | 12466851 |
|  | 12697810 |
|  | 12130649 |
|  | 12637525 |
|  | 12730054 |
|  | 14769808 |
|  | 1347949 |
|  | 15890751 |
|  | 15170225 |
|  | 16510265 |
|  | 11217851 |
|  | 8632909 |
|  | 9792677 |
|  | 12540563 |
|  | 15721302 |
|  | 16950125 |
|  | 15210734 |
|  | 12562867 |
|  | 1922387 |
|  | 12947325 |
|  | 15721216 |
|  | 16492667 |
|  | 17005865 |
|  | 14966273 |
|  | 10570282 |
|  | 17464174 |
|  | 17427197 |
|  | 12349907 |
|  | 17131384 |
|  | 11134045 |
|  | 11994045 |
|  | 11884401 |
|  | 11844932 |
|  | 15917991 |
|  | 16944316 |
|  | 17297454 |
|  | 10648599 |
|  | 15262203 |
|  | 15582777 |
|  | 15923189 |
|  | 12629153 |
|  | 15574735 |
|  | 16943367 |
|  | 17337702 |
|  | 14675480 |
|  | 16916926 |
|  | 15243131 |
|  | 15880942 |
|  | 14622970 |
|  | 15502830 |
|  | 12082091 |
|  | 17464186 |
|  | 15985432 |
|  | 9628824 |
|  | 16954218 |
|  | 17548467 |
|  | 16321971 |
|  | 12935895 |
|  | 14568551 |
|  | 12538595 |
|  | 15256218 |
|  | 11805112 |
|  | 16317051 |
|  | 12727216 |
|  | 15829913 |
|  | 15964695 |
|  | 16047735 |
|  | 15899793 |
|  | 16278291 |
|  | 17458890 |
|  | 15347670 |
|  | 15078890 |
|  | 1319925 |
|  | 15489642 |
|  | 14671106 |
|  | 15899886 |
|  | 14714552 |
|  | 12393899 |
|  | 16278684 |
|  | 16272159 |
|  | 17317670 |
|  | 16291755 |
|  | 17522155 |
|  | 16621802 |
|  | 14636584 |
|  | 14522949 |
|  | 14988413 |
|  | 16906226 |
|  | 15937334 |
|  | 16538383 |
|  | 11841548 |
|  | 17142261 |
|  | 11961297 |
|  | 17237147 |
|  | 9421269 |
|  | 12469121 |
|  | 1712480 |
|  | 15249224 |
|  | 16373414 |
|  | 11307167 |
|  | 7592886 |
|  | 11042159 |
|  | 17267664 |
|  | 11952164 |
|  | 16141211 |
|  | 16116448 |
|  | 14688221 |
|  | 11409852 |
|  | 16979915 |
|  | 16139272 |
|  | 12081562 |
|  | 10574913 |
|  | 15691764 |
|  | 15118098 |
|  | 16280323 |
|  | 17574041 |
|  | 15572359 |
|  | 15739119 |
|  | 16149052 |
|  | 14576154 |
|  | 16617096 |
|  | 15071597 |
|  | 17363453 |
|  | 11356865 |
|  | 15616583 |
|  | 9621077 |
|  | 17333127 |
|  | 12050114 |
|  | 15040022 |
|  | 17149612 |
|  | 15991247 |
|  | 12773577 |
|  | 14605008 |
|  | 8444355 |
|  | 16888810 |
|  | 17015703 |
|  | 11937531 |
|  | 17309799 |
|  | 15371543 |
|  | 15967991 |
|  | 12072567 |
|  | 14702039 |
|  | 16805921 |
|  | 8208531 |
|  | 17410437 |
|  | 8152805 |
|  | 11832339 |
|  | 12800247 |
|  | 16887830 |
|  | 17582742 |
|  | 17512932 |
|  | 16857343 |
|  | 16978658 |
|  | 7619503 |
|  | 15229233 |
|  | 12584202 |
|  | 12832284 |
|  | 16514068 |
|  | 16287813 |
|  | 10051434 |
|  | 16990258 |
|  | 14670631 |
|  | 8794306 |
|  | 15364926 |
|  | 11131153 |
|  | 8626571 |
|  | 15327774 |
|  | 16432225 |
|  | 9060415 |
|  | 1846291 |
|  | 16941494 |
|  | 16705745 |
|  | 12606501 |
|  | 17074386 |
|  | 12134156 |
|  | 16714296 |
|  | 16837555 |
|  | 17256754 |
|  | 12477973 |
|  | 15030409 |
|  | 15321994 |
|  | 15030407 |
|  | 12603851 |
|  | 16794828 |
|  | 17107998 |
|  | 12661038 |
|  | 10206983 |
|  | 12637339 |
|  | 15731359 |
|  | 16081426 |
|  | 12623863 |
|  | 16914058 |
|  | 16730415 |
|  | 11702783 |
|  | 12718435 |
|  | 12551961 |
|  | 11583024 |
|  | 12482669 |
|  | 14587029 |
|  | 16887817 |
|  | 15563472 |
|  | 17274723 |
|  | 15489334 |
|  | 17212589 |
|  | 12538349 |
|  | 17513610 |
|  | 15247230 |
|  | 12393886 |
|  | 8290275 |
|  | 16814421 |
|  | 15030400 |
|  | 12388422 |
|  | 12388423 |
|  | 11260070 |
|  | 17088403 |
|  | 16510590 |
|  | 17417601 |
|  | 15128760 |
|  | 15811423 |
|  | 16687572 |
|  | 17490811 |
|  | 15737734 |
|  | 16049186 |
|  | 10559944 |
|  | 12700162 |
|  | 16707097 |
|  | 12529294 |
|  | 11889128 |
|  | 16239230 |
|  | 12705474 |
|  | 12555071 |
|  | 16106045 |
|  | 16795048 |
|  | 17113581 |
|  | 9792705 |
|  | 16399079 |
|  | 7592946 |
|  | 15273746 |
|  | 10912793 |
|  | 12535947 |
|  | 12854829 |
|  | 10551817 |
|  | 16611738 |
|  | 17068135 |
|  | 17077290 |
|  | 12456688 |
|  | 10748187 |
|  | 17396120 |
|  | 12960100 |
|  | 12736212 |
|  | 17382881 |
|  | 17068339 |
|  | 17507094 |
|  | 17371162 |
|  | 12943720 |
|  | 14597424 |
|  | 16670284 |
|  | 11726647 |
|  | 16808899 |
|  | 14960303 |
|  | 14963006 |
|  | 17442737 |
|  | 12223545 |
|  | 16867183 |
|  | 17107949 |
|  | 1716439 |
|  | 15305288 |
|  | 14651992 |
|  | 17311928 |
|  | 14673008 |
|  | 12095152 |
|  | 17215072 |
|  | 7535768 |
|  | 12032872 |
|  | 11682603 |
|  | 12161450 |
|  | 17068343 |
|  | 12612072 |
|  | 11923482 |
|  | 8108135 |
|  | 11891214 |
|  | 15686869 |
|  | 15975997 |
|  | 7797459 |
|  | 15655348 |
|  | 12477932 |
|  | 10349636 |
|  | 16944453 |
|  | 12081892 |
|  | 11923207 |
|  | 16467359 |
|  | 16966488 |
|  | 12925581 |
|  | 12699904 |
|  | 11923300 |
|  | 16926156 |
|  | 17522999 |
|  | 15355986 |
|  | 16670094 |
|  | 11866540 |
|  | 17396111 |
|  | 11866440 |
|  | 12821662 |
|  | 9371744 |
|  | 14501944 |
|  | 16242072 |
|  | 15051723 |
|  | 17442811 |
|  | 12042309 |
|  | 14605001 |
|  | 11278799 |
|  | 17418417 |
|  | 8939914 |
|  | 17543571 |
|  | 17504381 |
|  | 16226508 |
|  | 12446729 |
|  | 12937136 |
|  | 17499702 |
|  | 15975921 |
|  | 11830463 |
|  | 15901735 |
|  | 14575703 |
|  | 1540184 |
|  | 17583675 |
|  | 15059773 |
|  | 12832467 |
|  | 17341442 |
|  | 12473660 |
|  | 9779826 |
|  | 17709754 |
|  | 7997269 |
|  | 15617763 |
|  | 17496918 |
|  | 15383658 |
|  | 16844778 |
|  | 12097372 |
|  | 17016429 |
|  | 15748847 |
|  | 11741977 |
|  | 16020276 |
|  | 8626767 |
|  | 17443665 |
|  | 15125795 |
|  | 7687743 |
|  | 16641227 |
|  | 15121188 |
|  | 16624816 |
|  | 15922485 |
|  | 15113754 |
|  | 11076861 |
|  | 10635328 |
|  | 17314319 |
|  | 11799072 |
|  | 17303384 |
|  | 10224087 |
|  | 15026121 |
|  | 8552640 |
|  | 17303382 |
|  | 9149902 |
|  | 11773377 |
|  | 9558095 |
|  | 16863646 |
|  | 12663502 |
|  | 12930843 |
|  | 16893669 |
|  | 16081808 |
|  | 15757891 |
|  | 17525735 |
|  | 15784503 |
|  | 15746430 |
|  | 14566055 |
|  | 15336571 |
|  | 12072413 |
|  | 14602725 |
|  | 15282307 |
|  | 12097495 |
|  | 15797923 |
|  | 15069082 |
|  | 15107845 |
|  | 12920127 |
|  | 17351113 |
|  | 15365619 |
|  | 15016620 |
|  | 15537893 |
|  | 12689928 |
|  | 9415407 |
|  | 15068691 |
|  | 12183542 |
|  | 16644735 |
|  | 17192257 |
|  | 16998804 |
|  | 17081988 |
|  | 15257182 |
|  | 16641100 |
|  | 15020650 |
|  | 14502223 |
|  | 17081983 |
|  | 15166036 |
|  | 17628608 |
|  | 16283431 |
|  | 17294242 |
|  | 15544353 |
|  | 16344560 |
|  | 1717989 |
|  | 15052686 |
|  | 17478078 |
|  | 15471881 |
|  | 12872135 |
|  | 17149706 |
|  | 15537896 |
|  | 11668049 |
|  | 17237423 |
|  | 16319118 |
|  | 14593093 |
|  | 15132979 |
|  | 14634061 |
|  | 16737746 |
|  | 16820791 |
|  | 12362243 |
|  | 10982368 |
|  | 16207820 |
|  | 14552884 |
|  | 16326014 |
|  | 11789669 |
|  | 14715940 |
|  | 15545519 |
|  | 16820883 |
|  | 16735500 |
|  | 17035635 |
|  | 15498890 |
|  | 14634064 |
|  | 12388473 |
|  | 15695173 |
|  | 15072963 |
|  | 7836374 |
|  | 17239373 |
|  | 15728253 |
|  | 16595703 |
|  | 16515552 |
|  | 10922068 |
|  | 16126730 |
|  | 17583725 |
|  | 17695723 |
|  | 1437147 |
|  | 15951569 |
|  | 14711824 |
|  | 15677498 |
|  | 16614081 |
|  | 14532121 |
|  | 16973750 |
|  | 12167619 |
|  | 12223406 |
|  | 12794156 |
|  | 16414009 |
|  | 14580718 |
|  | 12955074 |
|  | 12441075 |
|  | 11986622 |
|  | 16897752 |
|  | 15537391 |
|  | 11930938 |
|  | 12531514 |
|  | 11044099 |
|  | 14749824 |
|  | 12782152 |
|  | 11741541 |
|  | 15203192 |
|  | 14741046 |
|  | 12435482 |
|  | 16126724 |
|  | 15921682 |
|  | 12132588 |
|  | 17482686 |
|  | 16135819 |
|  | 12217325 |
|  | 16849642 |
|  | 12491158 |
|  | 16156742 |
|  | 9087672 |
|  | 12647296 |
|  | 16556602 |
|  | 16217033 |
|  | 12871576 |
|  | 16407894 |
|  | 8226933 |
|  | 10074203 |
|  | 11756405 |
|  | 12121979 |
|  | 9223324 |
|  | 7939721 |
|  | 12663469 |
|  | 16504549 |
|  | 15573401 |
|  | 12213812 |
|  | 16368932 |
|  | 15158083 |
|  | 11687663 |
|  | 12849998 |
|  | 9733512 |
|  | 16050810 |
|  | 16972271 |
|  | 12643788 |
|  | 12663671 |
|  | 17560563 |
|  | 11792123 |
|  | 17265428 |
|  | 16831126 |
|  | 11160759 |
|  | 15650391 |
|  | 16627622 |
|  | 17502994 |
|  | 15456767 |
|  | 14984009 |
|  | 15879168 |
|  | 15242988 |
|  | 16820947 |
|  | 17183546 |
|  | 15201339 |
|  | 16332685 |
|  | 17662006 |
|  | 9857190 |
|  | 14515181 |
|  | 15569628 |
|  | 16574793 |
|  | 16569214 |
|  | 11776327 |
|  | 1327976 |
|  | 16699726 |
|  | 15735702 |
|  | 15143154 |
|  | 14597699 |
|  | 15642734 |
|  | 15907834 |
|  | 9535927 |
|  | 12880367 |
|  | 16533948 |
|  | 15542825 |
|  | 12006386 |
|  | 15292176 |
|  | 15322023 |
|  | 10558995 |
|  | 14701865 |
|  | 16044092 |
|  | 14701866 |
|  | 15143165 |
|  | 14718391 |
|  | 12062026 |
|  | 16782007 |
|  | 15297310 |
|  | 10958792 |
|  | 15964803 |
|  | 10627507 |
|  | 10825211 |
|  | 12952860 |
|  | 16406609 |
|  | 15509801 |
|  | 16141073 |
|  | 16141072 |
|  | 15169786 |
|  | 12057008 |
|  | 14676217 |
|  | 16995340 |
|  | 17065231 |
|  | 16796978 |
|  | 17510316 |
|  | 11960991 |
|  | 15653554 |
|  | 12030372 |
|  | 12645629 |
|  | 14638694 |
|  | 9155018 |
|  | 11409918 |
|  | 16813525 |
|  | 11684667 |
|  | 16934944 |
|  | 14506249 |
|  | 17209045 |
|  | 16024175 |
|  | 12676937 |
|  | 15064239 |
|  | 16004581 |
|  | 16482086 |
|  | 16554472 |
|  | 16273281 |
|  | 17308007 |
|  | 17198696 |
|  | 10799874 |
|  | 16723512 |
|  | 16723511 |
|  | 15950773 |
|  | 15809051 |
|  | 2164259 |
|  | 8896447 |
|  | 15814736 |
|  | 12068299 |
|  | 70317 |
|  | 17600551 |
|  | 17273789 |
|  | 14744867 |
|  | 16040801 |
|  | 17029782 |
|  | 12810719 |
|  | 17620366 |
|  | 15173068 |
|  | 17072965 |
|  | 16253958 |
|  | 16209712 |
|  | 10509564 |
|  | 15322069 |
|  | 15572037 |
|  | 10402467 |
|  | 15629424 |
|  | 12921749 |
|  | 14985328 |
|  | 16691571 |
|  | 8639570 |
|  | 12810726 |
|  | 16434965 |
|  | 17641779 |
|  | 16039615 |
|  | 11875120 |
|  | 17022980 |
|  | 15964902 |
|  | 12682854 |
|  | 12690112 |
|  | 11756441 |
|  | 16877348 |
|  | 11114188 |
|  | 15919053 |
|  | 15249196 |
|  | 17388789 |

---

|  |  |
| --- | --- |
| Organism | Homo sapiens {Organism urn:agi-taxid:9606} |
|  | Mus musculus {Organism urn:agi-taxid:10090} |
|  | Rattus norvegicus {Organism urn:agi-taxid:10116} |
|  | Homo sapiens |
|  | Mus musculus |
|  | Rattus norvegicus |
|  | Danio rerio |
|  | Bos taurus |

---

|  |  |
| --- | --- |
| Mouse chromosome position | 7 69.25 cM |
|  | 7 61.0 cM |

---

|  |  |
| --- | --- |
| OMIM ID | 601795 |
|  | 61408 |
|  | OMIM:601795 |

---

|  |  |
| --- | --- |
| Rat chromosome position | 1q36 |

---

|  |  |
| --- | --- |
| Hugo ID | 6877 |
|  | HGNC:6877 |

---

|  |  |
| --- | --- |
| Human chromosome position | 16p11.2 |

---

|  |  |
| --- | --- |
| Swiss-Prot Accession | P27361 |
|  | Q9BWJ1 |
|  | L7RXH5 |
|  | P27361.4 |
|  | Q63844 |
|  | Q63844.5 |
|  | P21708 |
|  | P21708.5 |
|  | A8CZ58 |
|  | B0LPG3 |
|  | Q8NHX1 |
|  | Q61531 |
|  | Q8K0X5 |
|  | Q91YW5 |
|  | Q4PIY8 |
|  | Q62686 |
|  | Q9JJ13 |
|  | Q7Z3H5 |
|  | Q8NHX0 |
|  | Q3U2H3 |
|  | Q8NHX1\_HUMAN |
|  | MK03\_RAT |
|  | A8CZ58\_HUMAN |
|  | B0LPG3\_HUMAN |
|  | MK03\_HUMAN |
|  | MK03\_MOUSE |

---

|  |  |
| --- | --- |
| PIR ID | A48082 |
|  | S28184 |
|  | JC1451 |

---

|  |  |
| --- | --- |
| GenBank ID | NC\_000016 |
|  | XR\_243293 |
|  | NM\_001109891 |
|  | NP\_001103361 |
|  | NM\_002746 |
|  | NP\_002737 |
|  | NM\_001040056 |
|  | NP\_001035145 |
|  | NG\_029936 |
|  | AC012645 |
|  | AC243947 |
|  | AJ222708 |
|  | CH471238 |
|  | EAW79912 |
|  | EAW79913 |
|  | EAW79914 |
|  | EAW79915 |
|  | EAW79916 |
|  | EAW79917 |
|  | EU332853 |
|  | ABY87542 |
|  | HC462820 |
|  | CBK51145 |
|  | JX512450 |
|  | AGC09597 |
|  | AK091009 |
|  | BAG52261 |
|  | AK307995 |
|  | AY033607 |
|  | AAK52329 |
|  | AY033608 |
|  | AAK52330 |
|  | BC000205 |
|  | AAH00205 |
|  | BC013992 |
|  | AAH13992 |
|  | BX537897 |
|  | CAD97888 |
|  | DA351318 |
|  | DQ399291 |
|  | ABD60302 |
|  | KJ534878 |
|  | AHW56518 |
|  | M84490 |
|  | AAA36142 |
|  | X60188 |
|  | CAA42744 |
|  | Z11696 |
|  | CAA77754 |
|  | P27361 |
|  | NC\_000073 |
|  | NM\_011952 |
|  | NP\_036082 |
|  | AC124505 |
|  | X90997 |
|  | AK155287 |
|  | BAE33167 |
|  | BC013754 |
|  | AAH13754 |
|  | BC029712 |
|  | AAH29712 |
|  | BQ929318 |
|  | S58470 |
|  | AAB19973 |
|  | X64605 |
|  | CAA45889 |
|  | Z14249 |
|  | Q63844 |
|  | NC\_005100 |
|  | NM\_017347 |
|  | NP\_059043 |
|  | AC\_000069 |
|  | AABR07005779 |
|  | AAHX01008512 |
|  | AF155236 |
|  | AAF71666 |
|  | M38194 |
|  | AAA41123 |
|  | M61177 |
|  | AAA63486 |
|  | S46779 |
|  | AAA11604 |
|  | U05219 |
|  | U12008 |
|  | AAA20009 |
|  | X65198 |
|  | CAA46318 |
|  | P21708 |
|  | NC\_018927 |
|  | DQ893440 |
|  | ABM84366 |
|  | DQ896778 |
|  | ABM87777 |
|  | AC\_000029 |
|  | AAHY01066409 |
|  | AC\_000148 |
|  | ABBA01013103 |
|  | AABR06008868 |
|  | NT\_010393 |
|  | NW\_001838235 |
|  | NW\_926539 |
|  | AC\_000059 |
|  | CR595737 |
|  | CR596064 |
|  | CR597147 |
|  | CR600245 |
|  | CR603463 |
|  | CR606522 |
|  | CR607536 |
|  | CR620058 |
|  | CR621455 |
|  | CR621688 |
|  | CR621977 |
|  | Q7Z3H5 |
|  | Q8NHX0 |
|  | Q8NHX1 |
|  | Q9BWJ1 |
|  | NT\_039433 |
|  | NW\_001030877 |
|  | Q3U2H3 |
|  | NW\_047562 |
|  | NW\_001084773 |
|  | XM\_001079997 |
|  | XP\_001079997 |
|  | 204052 |
|  | 204051 |
|  | 204054 |
|  | 204053 |
|  | 258470 |
|  | 557882 |
|  | 38257141 |
|  | 31220 |
|  | 31221 |
|  | 38257140 |
|  | BI414398 |
|  | 124000537 |
|  | 12652898 |
|  | 12652899 |
|  | 20988620 |
|  | 21749281 |
|  | AW128045 |
|  | 56627 |
|  | 547569 |
|  | 56626 |
|  | 20988619 |
|  | XP\_686128 |
|  | 123787473 |
|  | NT\_089315 |
|  | ZDB-GENE-030131-5613 |
|  | 52001483 |
|  | 50858 |
|  | 50859 |
|  | 23882 |
|  | Dr.113689 |
|  | NC\_007326 |
|  | XM\_681036 |
|  | 74759131 |
|  | 3273090 |
|  | 8050445 |
|  | 8050444 |
|  | 8393330 |
|  | 8393331 |
|  | 236372 |
|  | 20384794 |
|  | 20384796 |
|  | 236373 |
|  | 20384795 |
|  | 20384797 |
|  | XM\_914793 |
|  | 31873913 |
|  | 31873912 |
|  | 34860310 |
|  | 123993529 |
|  | 21489933 |
|  | 74760366 |
|  | 21489932 |
|  | 15559271 |
|  | 15559270 |
|  | 125804488 |
|  | dre:557882 |
|  | 51480188 |
|  | 51472974 |
|  | 115311606 |
|  | 984670 |
|  | XP\_919886 |
|  | Q4PIY8 |
|  | 186696 |
|  | 1335009 |
|  | 186695 |
|  | 119600318 |
|  | 119600319 |
|  | 74717576 |
|  | BG075319 |
|  | 232066 |
|  | 119600322 |
|  | 119600323 |
|  | 119600320 |
|  | 119600321 |
|  | 515499 |
|  | 515498 |
|  | AW116014 |
|  | XP\_609884 |
|  | 74199274 |
|  | 15489307 |
|  | 15489308 |
|  | 51762371 |
|  | NC\_007112 |
|  | 126002739 |
|  | NW\_001511913 |
|  | XM\_609884 |
|  | 74760367 |
|  | NW\_001494303 |
|  | 232067 |

---

|  |  |
| --- | --- |
| Swiss-Prot ID | MK03\_HUMAN |
|  | MK03\_MOUSE |
|  | MK03\_RAT |
|  | Q8NHX1\_HUMAN |
|  | A8CZ58\_HUMAN |
|  | Q9BWJ1\_HUMAN |
|  | B0LPG3\_HUMAN |

---

|  |  |
| --- | --- |
| Cell Localization | Cytoplasm |
|  | Nucleus |
|  | Membrane |
|  | Caveola |
|  | Plasma membrane |

---

|  |  |
| --- | --- |
| Ensembl ID | ENSG00000102882 |
|  | ENSP00000378625.3 |
|  | ENST00000395199.7 |
|  | ENSP00000327293.5 |
|  | ENST00000322266.9 |
|  | ENSP00000263025.4 |
|  | ENST00000263025.9 |
|  | ENSMUSG00000063065 |
|  | ENSMUSP00000051619.9 |
|  | ENSMUST00000057669.15 |
|  | ENSRNOG00000053583 |
|  | ENSRNOP00000070784.1 |
|  | ENSRNOT00000087625.1 |
|  | ENSP00000378625 |
|  | ENST00000395199 |
|  | ENSP00000327293 |
|  | ENST00000322266 |
|  | ENSP00000263025 |
|  | ENST00000263025 |
|  | ENSMUSP00000051619 |
|  | ENSMUST00000057669 |
|  | ENSRNOP00000070784 |
|  | ENSRNOT00000087625 |
|  | ENSRNOG00000019601 |
|  | ENSRNOP00000026627 |
|  | ENSRNOT00000026627 |

---

|  |  |
| --- | --- |
| MGI ID | MGI:1346859 |
|  | 1346859 |
|  | 105920 |
|  | 97176 |

---

|  |  |
| --- | --- |
| RGD ID | 3046 |
|  | 61408 |

---

|  |  |
| --- | --- |
| Unigene ID | Hs.861 |
|  | Mm.8385 |
|  | Rn.2592 |
|  | Dr.113689 |
|  | Bt.5687 |

---

|  |  |
| --- | --- |
| Homologene ID | 55682 |

---

|  |  |
| --- | --- |
| Shape | Sickle-vertex |

---

|  |  |
| --- | --- |
| IPI ID | IPI00018195 |
|  | IPI00206172 |
|  | IPI00231081 |
|  | IPI00230277 |
|  | IPI00742900 |
|  | IPI00304111 |
|  | IPI00984821 |
|  | IPI00793141 |
|  | IPI00654328 |
|  | IPI00750828 |
|  | IPI00776948 |

---

|  |  |
| --- | --- |
| Microarray ID | 1427060\_at |
|  | 101834\_at |

---

|  |  |
| --- | --- |
| Homo sapiens Chromosome position | 16p11.2 |
|  | 16p12-p11.2 |

---

|  |  |
| --- | --- |
| Ensembl Transcript ID | ENST00000263025 |
|  | ENST00000403394 |
|  | ENST00000322266 |
|  | ENST00000395199 |
|  | ENST00000395202 |

---

|  |  |
| --- | --- |
| ENSEMBL | ENSG00000102882 |

---

|  |  |
| --- | --- |
| FunctionalClass | protein kinase |
|  | kinase |
|  | MAP kinase |
|  | protein binding |
|  | protein-tyrosine kinase |
|  | protein kinase CK2 |
|  | phosphotyrosine binding |
|  | nucleotide binding |
|  | protein serine/threonine kinase |
|  | transferase |
|  | ATP binding |
|  | cAMP-dependent protein kinase |

---

|  |  |
| --- | --- |
| Hugo Symbol | MAPK3 |

---

|  |  |
| --- | --- |
| Bos taurus Chromosome position | 25 |

---

|  |  |
| --- | --- |
| Mus musculus Chromosome position | 7 61.0 cM |

---

|  |  |
| --- | --- |
| KEGG pathway | Chronic myeloid leukemia |
|  | Gap junction |
|  | Colorectal cancer |
|  | Melanoma |
|  | Melanogenesis |
|  | Natural killer cell mediated cytotoxicity |
|  | Focal adhesion |
|  | Long-term potentiation |
|  | Dorso-ventral axis formation |
|  | ErbB signaling pathway |
|  | Endometrial cancer |
|  | Glioma |
|  | GnRH signaling pathway |
|  | Long-term depression |
|  | Renal cell carcinoma |
|  | Non-small cell lung cancer |
|  | Axon guidance |
|  | Pancreatic cancer |
|  | MAPK signaling pathway |
|  | TGF-beta signaling pathway |
|  | mTOR signaling pathway |
|  | VEGF signaling pathway |
|  | Adherens junction |
|  | Thyroid cancer |
|  | Insulin signaling pathway |
|  | Type II diabetes mellitus |
|  | Regulation of actin cytoskeleton |
|  | Fc epsilon RI signaling pathway |

---

|  |  |
| --- | --- |
| ProteinAtlas SubcellularLocation | Main location: Nucleus but not nucleoli; Other location: Cytoskeleton (Microtubules); Expression type: Staining; Reliability: Uncertain |

---

|  |  |
| --- | --- |
| Rattus norvegicus Chromosome position | 1q36 |

---

|  |  |
| --- | --- |
| Ensembl Gene ID | ENSG00000102882 |

---

|  |  |
| --- | --- |
| ProteinAtlas NormalTissue | Tissue: adrenal gland; Cell type: glandular cells; Level: Medium; Expression type: APE; Reliability: Medium |
|  | Tissue: appendix; Cell type: glandular cells; Level: Medium; Expression type: APE; Reliability: Medium |
|  | Tissue: appendix; Cell type: lymphoid tissue; Level: Low; Expression type: APE; Reliability: Medium |
|  | Tissue: bone marrow; Cell type: hematopoietic cells; Level: Medium; Expression type: APE; Reliability: Medium |
|  | Tissue: breast; Cell type: glandular cells; Level: Medium; Expression type: APE; Reliability: Medium |
|  | Tissue: bronchus; Cell type: respiratory epithelial cells; Level: High; Expression type: APE; Reliability: Medium |
|  | Tissue: cerebellum; Cell type: cells in granular layer; Level: Medium; Expression type: APE; Reliability: Medium |
|  | Tissue: cerebellum; Cell type: cells in molecular layer; Level: Low; Expression type: APE; Reliability: Medium |
|  | Tissue: cerebellum; Cell type: Purkinje cells; Level: High; Expression type: APE; Reliability: Medium |
|  | Tissue: cerebral cortex; Cell type: glial cells; Level: Medium; Expression type: APE; Reliability: Medium |
|  | Tissue: cerebral cortex; Cell type: neuronal cells; Level: Medium; Expression type: APE; Reliability: Medium |
|  | Tissue: cervix, uterine; Cell type: glandular cells; Level: Medium; Expression type: APE; Reliability: Medium |
|  | Tissue: cervix, uterine; Cell type: squamous epithelial cells; Level: Medium; Expression type: APE; Reliability: Medium |
|  | Tissue: colon; Cell type: glandular cells; Level: High; Expression type: APE; Reliability: Medium |
|  | Tissue: duodenum; Cell type: glandular cells; Level: Medium; Expression type: APE; Reliability: Medium |
|  | Tissue: epididymis; Cell type: glandular cells; Level: Medium; Expression type: APE; Reliability: Medium |
|  | Tissue: esophagus; Cell type: squamous epithelial cells; Level: Medium; Expression type: APE; Reliability: Medium |
|  | Tissue: fallopian tube; Cell type: glandular cells; Level: High; Expression type: APE; Reliability: Medium |
|  | Tissue: gall bladder; Cell type: glandular cells; Level: Medium; Expression type: APE; Reliability: Medium |
|  | Tissue: heart muscle; Cell type: myocytes; Level: Medium; Expression type: APE; Reliability: Medium |
|  | Tissue: hippocampus; Cell type: glial cells; Level: Medium; Expression type: APE; Reliability: Medium |
|  | Tissue: hippocampus; Cell type: neuronal cells; Level: High; Expression type: APE; Reliability: Medium |
|  | Tissue: kidney; Cell type: cells in glomeruli; Level: Medium; Expression type: APE; Reliability: Medium |
|  | Tissue: kidney; Cell type: cells in tubules; Level: Medium; Expression type: APE; Reliability: Medium |
|  | Tissue: lateral ventricle; Cell type: glial cells; Level: Low; Expression type: APE; Reliability: Medium |
|  | Tissue: lateral ventricle; Cell type: neuronal cells; Level: High; Expression type: APE; Reliability: Medium |
|  | Tissue: liver; Cell type: bile duct cells; Level: Low; Expression type: APE; Reliability: Medium |
|  | Tissue: liver; Cell type: hepatocytes; Level: Low; Expression type: APE; Reliability: Medium |
|  | Tissue: lung; Cell type: macrophages; Level: Medium; Expression type: APE; Reliability: Medium |
|  | Tissue: lung; Cell type: pneumocytes; Level: Medium; Expression type: APE; Reliability: Medium |
|  | Tissue: lymph node; Cell type: germinal center cells; Level: Medium; Expression type: APE; Reliability: Medium |
|  | Tissue: lymph node; Cell type: non-germinal center cells; Level: Medium; Expression type: APE; Reliability: Medium |
|  | Tissue: nasopharynx; Cell type: respiratory epithelial cells; Level: High; Expression type: APE; Reliability: Medium |
|  | Tissue: oral mucosa; Cell type: squamous epithelial cells; Level: Medium; Expression type: APE; Reliability: Medium |
|  | Tissue: ovary; Cell type: follicle cells; Level: Medium; Expression type: APE; Reliability: Medium |
|  | Tissue: ovary; Cell type: ovarian stroma cells; Level: Low; Expression type: APE; Reliability: Medium |
|  | Tissue: pancreas; Cell type: exocrine glandular cells; Level: High; Expression type: APE; Reliability: Medium |
|  | Tissue: pancreas; Cell type: islets of Langerhans; Level: High; Expression type: APE; Reliability: Medium |
|  | Tissue: parathyroid gland; Cell type: glandular cells; Level: Medium; Expression type: APE; Reliability: Medium |
|  | Tissue: placenta; Cell type: decidual cells; Level: Medium; Expression type: APE; Reliability: Medium |
|  | Tissue: placenta; Cell type: trophoblastic cells; Level: High; Expression type: APE; Reliability: Medium |
|  | Tissue: prostate; Cell type: glandular cells; Level: Medium; Expression type: APE; Reliability: Medium |
|  | Tissue: rectum; Cell type: glandular cells; Level: Medium; Expression type: APE; Reliability: Medium |
|  | Tissue: salivary gland; Cell type: glandular cells; Level: Medium; Expression type: APE; Reliability: Medium |
|  | Tissue: seminal vesicle; Cell type: glandular cells; Level: High; Expression type: APE; Reliability: Medium |
|  | Tissue: skeletal muscle; Cell type: myocytes; Level: Medium; Expression type: APE; Reliability: Medium |
|  | Tissue: skin; Cell type: epidermal cells; Level: Medium; Expression type: APE; Reliability: Medium |
|  | Tissue: small intestine; Cell type: glandular cells; Level: Medium; Expression type: APE; Reliability: Medium |
|  | Tissue: smooth muscle; Cell type: smooth muscle cells; Level: Medium; Expression type: APE; Reliability: Medium |
|  | Tissue: spleen; Cell type: cells in red pulp; Level: Medium; Expression type: APE; Reliability: Medium |
|  | Tissue: spleen; Cell type: cells in white pulp; Level: Medium; Expression type: APE; Reliability: Medium |
|  | Tissue: stomach, lower; Cell type: glandular cells; Level: Medium; Expression type: APE; Reliability: Medium |
|  | Tissue: stomach, upper; Cell type: glandular cells; Level: Medium; Expression type: APE; Reliability: Medium |
|  | Tissue: testis; Cell type: cells in seminiferus ducts; Level: High; Expression type: APE; Reliability: Medium |
|  | Tissue: testis; Cell type: Leydig cells; Level: Medium; Expression type: APE; Reliability: Medium |
|  | Tissue: thyroid gland; Cell type: glandular cells; Level: Low; Expression type: APE; Reliability: Medium |
|  | Tissue: tonsil; Cell type: germinal center cells; Level: High; Expression type: APE; Reliability: Medium |
|  | Tissue: tonsil; Cell type: non-germinal center cells; Level: High; Expression type: APE; Reliability: Medium |
|  | Tissue: tonsil; Cell type: squamous epithelial cells; Level: High; Expression type: APE; Reliability: Medium |
|  | Tissue: urinary bladder; Cell type: urothelial cells; Level: High; Expression type: APE; Reliability: Medium |
|  | Tissue: uterus, post-menopause; Cell type: cells in endometrial stroma; Level: Low; Expression type: APE; Reliability: Medium |
|  | Tissue: uterus, post-menopause; Cell type: glandular cells; Level: Medium; Expression type: APE; Reliability: Medium |
|  | Tissue: uterus, pre-menopause; Cell type: cells in endometrial stroma; Level: Medium; Expression type: APE; Reliability: Medium |
|  | Tissue: uterus, pre-menopause; Cell type: glandular cells; Level: Medium; Expression type: APE; Reliability: Medium |
|  | Tissue: vagina; Cell type: squamous epithelial cells; Level: Medium; Expression type: APE; Reliability: Medium |
|  | Tissue: vulva/anal skin; Cell type: epidermal cells; Level: High; Expression type: APE; Reliability: Medium |

---

|  |  |
| --- | --- |
| LocatorString | pmquery:egfr+transactivation |
|  | pmftq:achondroplasia |
|  | gscquery:achondroplasia |

---

|  |  |
| --- | --- |
| Danio rerio Chromosome position | LG 3 |

---
